# Primary uterine non-Hodgkin’s lymphoma:a rare case report and review of the literature

**DOI:** 10.3389/fonc.2024.1462353

**Published:** 2024-12-18

**Authors:** Jing Wu, Ting Liu

**Affiliations:** Department of Radiology, the Affiliated Hospital of inner Mongolia Medical University, Hohhot, China

**Keywords:** non Hodgkin lymphoma (NHL), diffuse large b-cell lymphoma (DLBCL), diffusion weighted imaging, magnetic resonance (MR) imaging, primary uterine non-Hodgkin lymphoma (NHL)

## Abstract

Primary uterine non-Hodgkin lymphoma (NHL) is rarely reported, as its incidence is extremely low. We describe a 72 year old patient with primary uterine non-Hodgkin’s lymphoma stage IV, diffuse B-cell large cells, who responded well to cytotoxic chemotherapy (R-CHOP). Radiological investigations exhibited certain characteristics, including magnetic resonance T2 weighted imaging, enhanced scanning, diffusion weighted imaging and apparent diffusion coefficient values. The lesion in the anterior wall of the uterine body was relatively large, with a size of about 34mm×47mm×43mm. The gold standard for diagnosis is still the pathological examination of a biopsy specimen, which revealed diffuse large cell of B lineage. This article reviews data collected from 141 patients in the literature.

## Introduction

Diffuse large B-cell lymphoma (DLBCL) is an invasive and most common pathological type of non-Hodgkin lymphoma, accounting for approximately 30% to 40% of all cases. However, it is rare for the uterus and reproductive organs to be affected, which has not been well documented. Primary lymphomas of the female genital tract account for 0.2% to 1.1% of all extranodal lymphomas (1). Most cases of uterine lymphoma are caused by secondary involvement of the disease (2). Due to its rarity and non-specific clinical presentation, the diagnosis of uterine DLBCL is difficult. There are few studies at present, and therefore, there is currently no standard treatment for uterine DLBCL. However, several studies on DLBCL have investigated the efficacy of various treatment regimens, which were not specific to the female reproductive tract. For patients with DLBCL, the current standard treatment regimen is rituximab, doxorubicin, cyclophosphamide, vincristine and prednisone (R-CHOP), which has a cure rate of about 60%-70% and a relapse/refractory rate of 30%-40% (3, 4).

## Results

### Case report

**Figure 1:**
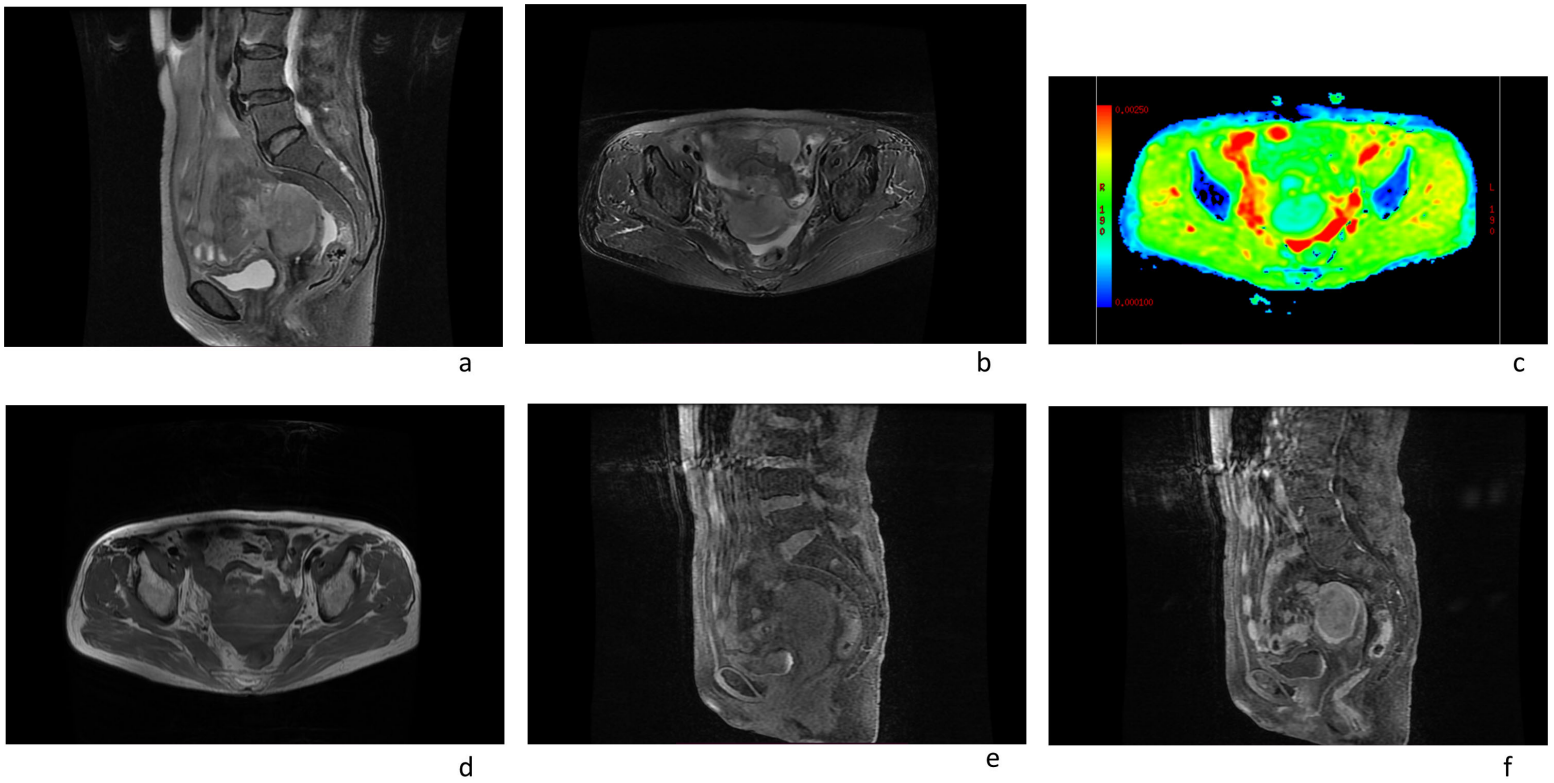
Magnetic resonance imaging scans of an 72-year-old female patient with a pelvic mass showed the following: **(A, B, D, E)** an enlarged uterus; **(F)** the arterial phase enhancement of the lesion was significantly uneven, the degree of venous phase enhancement was lower than that of the normal uterine myometrium. **(C)** The apparent diffusion coefficient (ADC) value of uterine mass was 0.751×10^-3^ mm^2^/s.

**Figure 2:**
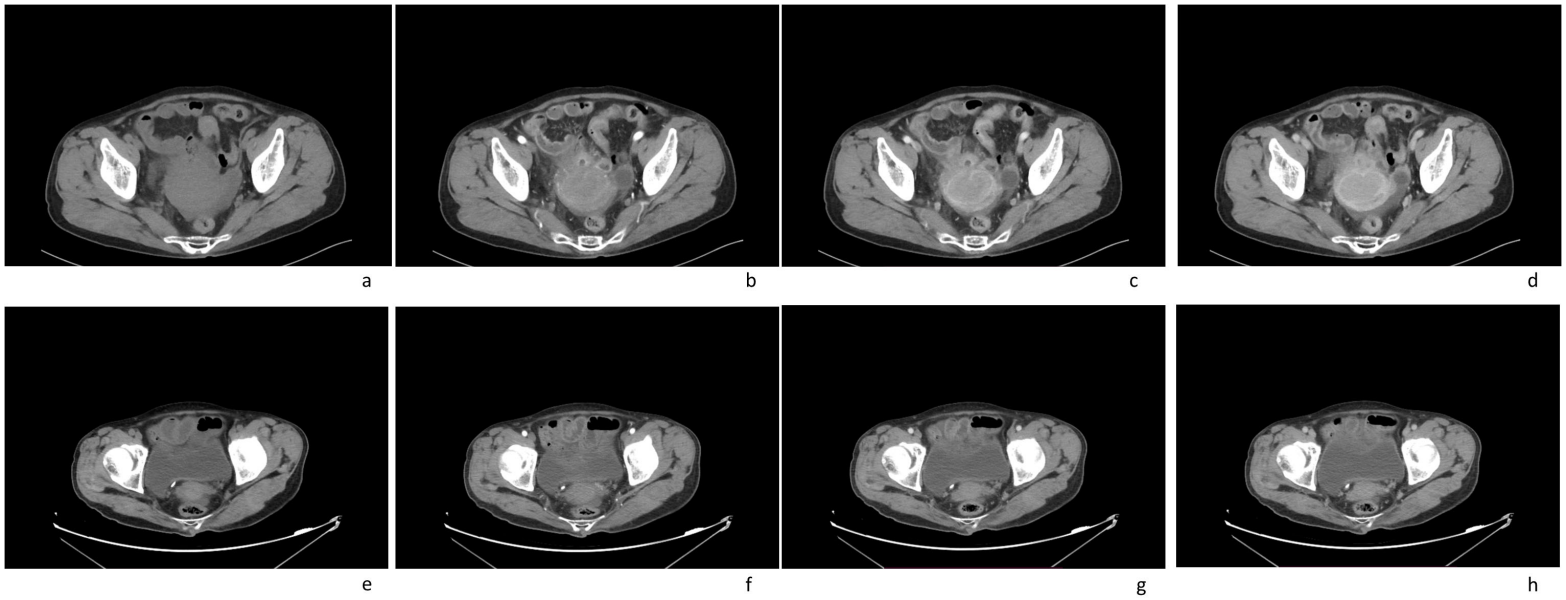
Computed tomography (CT) imaging scans of an 72-year-old female patient with a pelvic mass for more than 1 month: **(A)** plain CT imaging showed an enlarged uterine volume, soft tissue density shadow in the anterior wall of the uterus, with unclear boundary; **(B–D)** enhanced CT imaging(arterial phase[b]; venous phase[c]; delay phase[d]) showed that the lesion were slightly enhanced, the boundary was clear; **(E–H)** after treatment, enhanced CT imaging(arterial phase[f]; venous phase[g]; delay phase[h]) showed that the volume of the uterus had significantly reduced(plain CT imaging[e]).

**Figure 3:**
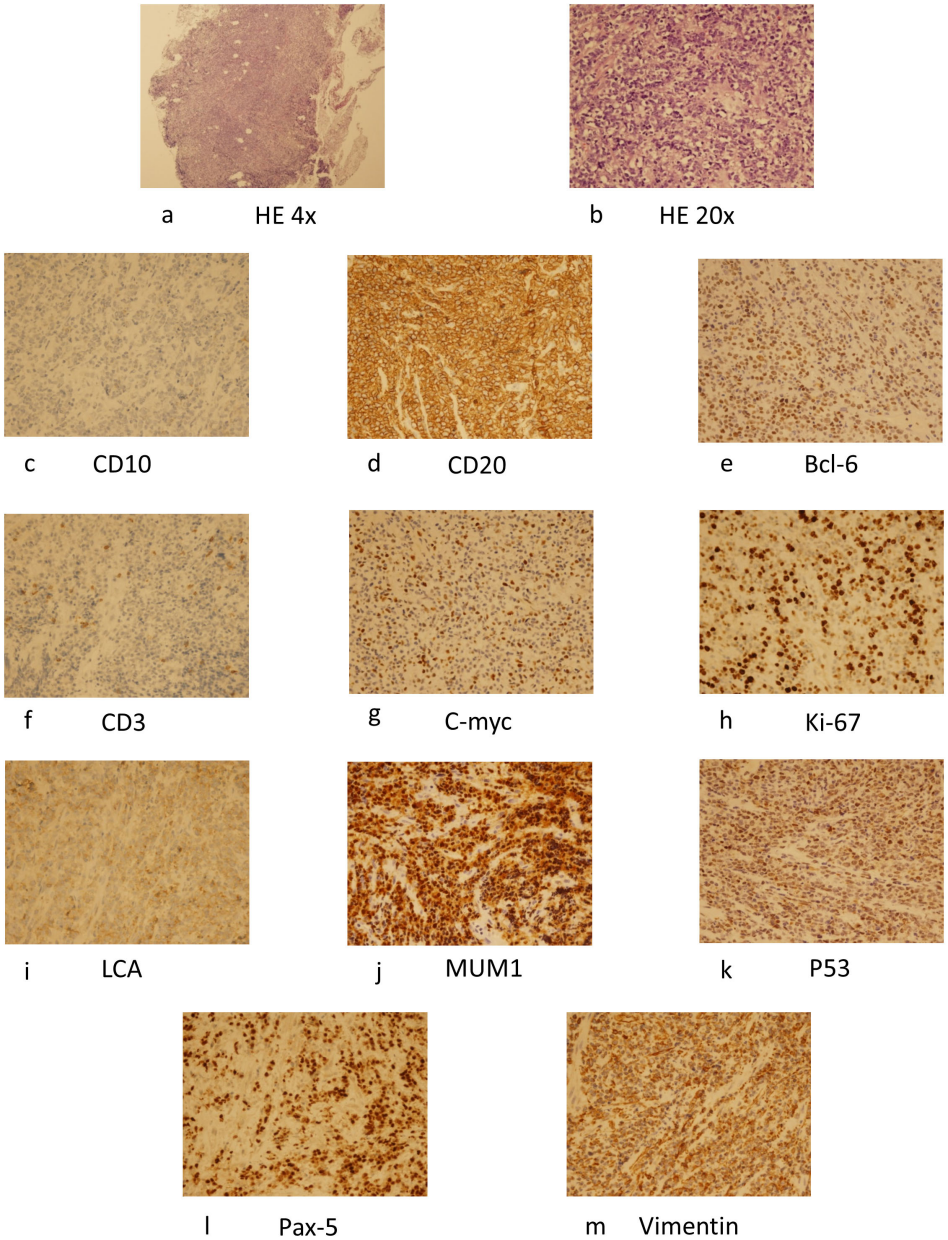
HE staining of the specimen (×40) **(A)**, (×200) **(B)**, and immunohistochemical examination revealed CD10 (+) **(C)**, CD20 (+) **(D)**, Bcl-6 (+) **(E)**, CD3(+) **(F)**, C-myc(+) **(G)**, Ki-67(+) **(H)**, LCA(+) **(I)**, MUM1(+) **(J)**, P53(+) **(K)**, Pax-5(+) **(L)**, Vimentin(+) **(M)** and suggested a diffuse large B-cell lymphoma. HE, hematoxylin.

In this case a 72-year-old woman experienced abdominal pain and urinary tract obstruction due to a large tumor in the uterus. The woman was in good general conditions had no hypertension, diabetes and dyslipidemia in her past medical History. Ultrasonography showed the presence of a large uterine mass, without clear evidence of local infiltration or lymphadenopathy. Magnetic resonance imaging (MRI) scan revealed that significant increase in uterine volume and slightly longer T1 and T2 signals were seen in the anterior wall and bottom of the uterine body. The lesion in the anterior wall of the uterine body was relatively large, with a size of about 34mm×47mm×43mm. After enhanced scanning, the arterial phase enhancement of the lesion was significantly uneven, the degree of venous phase enhancement was lower than that of the normal uterine myometrium, the boundary was clear, and the leading edge of the lesion was incomplete. Diffusion weighted imaging showed a significant high signal, which is due to the tight arrangement of tumor cells, small extracellular space, and limited cytoplasm, resulting in limited diffusion of water molecules inside and outside the cell. The apparent diffusion coefficient (ADC) value of 0.751×10^-3^ mm^2^/s was significantly decreased (**Figure 1**). Computed tomography (CT) showed that the uterine volume increased, soft tissue density shadow could be seen in the anterior wall of the uterus, with unclear boundary, the size was about 34mmx47mm, and the enhanced scan showed uneven enhancement, lower than the myometrium, and liquid density shadow could be seen in the pelvic cavity (**Figure 2**). An incisional biopsy was taken from the anterior wall of the uterus. Histological examination showed a diffuse large B-cell lymphoma of the uterus. Immunohistological examination revealed CD20(+), Bcl6(+), CD10(+),P40(-), CK7(-), P16(+), ER(-), PR(-), Vim(+), P53(mutant type, +), Ki67approximately 90%(+), HNF1-β(-), CK(-), WT1(-), NapsinA(-), PAX8(-), CA125(-), LCA(+), CD3(-), PAX5 (+), P504S(-), Desmin(-), Caldesmon(-), EBV(-), MUM(+), Bcl2(+) and c-Myc approximately 30-40%(+) suggested a diffuse large B-cell lymphoma from the uterus (**Figure 3**). A bone marrow biopsy was performed, and the results showed normal bone marrow cells without evidence of lymphoma involvement. The patient’s International Prognosric Index (IPI) score was 2. Under the supervision of the hematology department, a series of chemotherapy treatments including rituximab, cyclophosphamide, doxorubicin, vincristine, and prednisone (R-CHOP) were initiated. According to the National Comprehensive Cancer Network (NCCN) guideline, the patients in stage IV was planned to receive 6 cycles of RCHOP chemotherapy, and intrathecal methotrexate (IT-MTX) was given for Central Nervous System (CNS) prophylaxis, which were monitored every 3-6 monthly. A comparative CT scan was performed, and the results showed that after 3 courses of chemotherapy, all tumor locations significantly regressed. Before CT enhancement, the lesion density was relatively uniform, and obvious enhanced nodular shadow of about 6mm in diameter in the anterior wall of the uterus after the enhanced scan. The uterus is of normal shape and size. A small amount of fluid-like density shadow is seen in the pelvic cavity. Unfortunately, the patient died of heart failure after 3 cycles of RCHOP chemotherapy. The limitation is that interim positron emission tomography (PET)/CT scan was not done for the patient.

## Discussion

The table displays detailed information of 141 patients with primary uterine lymphoma (2, 10–63).

The uterine body, cervix, and vagina are rarely the origin sites of non-Hodgkin lymphoma (NHL). Primary Non-Hodgkin’s Lymphomas (NHL) of the uterus is a very rare disease, with non-specific clinical manifestations. Fox and More (4) defined three criteria for primary uterine lymphoma: (1) clinical limitation to the uterus; (2) There is no evidence of leukemia; (3) The interval between primary uterine lymphoma and secondary tumors is very long. If diagnosed with lymphoma, bone marrow biopsy must be performed to rule out leukemia.

Primary uterine and vaginal lymphoma usually present with non-specific symptoms such as abnormal vaginal or uterine bleeding, perineal discomfort, persistent vaginal discharge, abdominal pain, and urinary obstruction. These symptoms occur in many more diseases, such as cervical or endometrial carcinoma, adenomyosis, endometriosis and uterine fibroids, complicating the determination of a definitive differential diagnosis. The appearance of symptoms is not a characteristic of uterine lymphoma, therefore, making a correct diagnosis may take longer.

The age and pathological type of patients with uterine lymphoma are related, and diffuse large B-cell lymphoma is most common in women aged 35 to 45; Follicular lymphoma is more likely to occur in women over the age of 50 (1). Burkitt’s lymphoma usually occurs most often in children aged 5 and 10 (5). The current patients are elderly individuals with diffuse large B-cell lymphoma, which is relatively rare in clinical practice.

Magnetic resonance (MR) imaging is the most effective method for distinguishing lymphoma from cervical carcinoma, due to the fact that uterine DLBCL is a stromal rather than epithelial disease. The signal intensity of the uterine stroma and mucosa is usually intact and not invaded, which is different from cervical cancer. The Uterine lymphoma presents as a large mass within the uterus, and has similar signal intensity with that of other tumors, which is of low signal on T1 -weighted images, and high signal on T2 -weighted images. The contrast enhancement is common. The apparent diffusion coefficient values showed certain characteristics, which showed its ability to make a definitive differential diagnosis. The ADC of primary uterine lymphoma (0.696× 10^-3^ mm^2^/s) (6) was much lower than uterine fibroids and cervical cancer (1.15± 0.18× 10^-3^ mm^2^/s and 0.99± 0.18×10^-3^ mm^2^/s, respectively) (7).

CT is usually the preferred method for detecting and staging non Hodgkin lymphoma. The size and extent of the tumor can be accurately measured by CT. Moreover, CT can develop appropriate treatment plans and track treatment responses. CT can not only detect the lesions in the cervix, but also the involvement of the vagina, bladder, rectum and adjacent lymph nodes, which often presented as a large enhanced mass within the cervix, invasion of the vagina, bladder, rectum, and enlarged lymph nodes. No calcification is detected. CT is valuable for evaluating response to treatment, identifying active residual masses and assessing recurrence. Diagnosis invariably requires a core biopsy.

Currently, rituximab has been approved for monotherapy and when used in combination with CHOP, they are commonly referred to as R-CHOP (8). In several studies, the adding rituximab to CHOP therapy can improved complete response rate and prolonged event-free survival and overall survival (OS) in DLBCL patients (9) Szanto et al. found that using chemotherapy instead of radiotherapy can protect ovarian function, prevent micro metastasis (10). This has been proven to be effective in our patient.

In conclusion, elderly patients with primary uterine non Hodgkin lymphoma are relatively rare in clinical practice. When elderly patients develop pelvic masses, the physicians should consider the possibility of lymphoma and make a differential diagnosis.

## Data Availability

The original contributions presented in the study are included in the article/**Supplementary Material**. Further inquiries can be directed to the corresponding author/s.
