# Supplementary material for: Primary uterine non-Hodgkin’s lymphoma:a rare case report and review of the literature
Source: Front Oncol. 2024 Dec 18;14:1462353. doi: 10.3389/fonc.2024.1462353 (PMC11688220; doi:10.3389/fonc.2024.1462353)
Supplement: Supplementary file 1 [file Table1.docx]

Table Review of literature

S.N. Author (year) No Site Age, y (median) Hist Stage TX ref

1 Chorlton (1974) 13 Cx (6) 44 Histiocytic IE Only Sx (5) [11]

Vg (4) Only RT (2)

Ut (3) Only CT (1)

2 Harris (1984) 25 Cx (18) 43 DLC (17) IE (21) Only Sx (7) [12]

Vg (5) Nodular (7) IIE (3) Only RT (3)

Ut (2) Burkitts (1) IV (1) CT+RT (2)

Sx+RT/CT (13)

3 Komaki (1984) 3 Cx 38 DLC (2) IIE RT [13]

DMC (1)

4 Muntz (1991) 5 Cx 57 DLC (3) IE Sx+RT (3) [14]

DSC (1) RT (2)

Follicular (1)

5 Broekmans (1993) 1 Cx (polyp) 45 DLBC IE CT [15]

6 Kuo (1994) 1 Cx 40 DLBC IE Sx+CT [16]

7 Awwad (1994) 1 Cx+Vg 27 Large cell IE RT only [17]

8 Stroh (1995) 16 Cx (7) 59.5 DLBC (10) IE (5) CT+RT (12) [18]

Ut (4) small cell (4) IIE (7) OnlyCT (4)

large cell (2) IIIE (1)

IVA (1)

IVB (2)

9 Chandy(1998) 1 Cx (1) 50 DLBC IE CT+RT [19]

10 Lee (1998) 2 Cx 66 DLC IE Sx+RT [20]

11 Nasu (1998) 1 Cx 64 DLBC CT [21]

12 Grace(1999) 2 Cx 43.5 DLBC 3E [22]

IE

13 Cheong (2000) 1 Ut+Cx+ 62 Lymphoblastic IV Sx+CT [23]

Vg+BM Lymphoma

14 Vang (2000) 10 Cx (9) 55 DLBC (8) [24]

Follicular (1)

Zone B cell (1)

15 Pham (2003) 3 Cx (1) 66 DLBC IE Sx+RT (1) [25]

Ovary (1) Only CT (2)

Vg (1)

16 Venizelos (2003) 1 Cx 33 DLBC IV Only CT [26]

17 Gabriele (2003) 1 Cx+Vg 40 Sx+CT [27]

18 Au(2003) 1 Cx 45 DLC IE CT+RT [28]

19 Szantho (2003) 1 Cx+Vg 56 DLBC IE Sx+CT [10]

+pelvic side walls

20 Huseyin(2004) 1 Vg 30 DLBC IEA CT [29]

21 Chan (2005) 6 Cx 52 IE-2 [30]

IIIE-1

IVE-3

22 Garavaglia (2005) 3 CX+Vg 37 Large B cell IE-1 Sx+RT [31]

23 Heredia (2005) 2 Cx+Vg+LN 32 Diff mixed small IE CT+RT [32]

+large B cell

24 Dursun (2005) 1 Cx 51 DLBC Sx+CT [33]

25 Roopa(2006) 2 Cx+Ut 63.5 DLBC IE CT+RT (1)[34]

26 NOELLE(2006) 4 Cx 35 DLBC IIE CT [2]

Ut 56 DLBC IIE CT

Ut 43 MZL IIE CT+RT

Cx 49 DLBC IIE CT

27 Semczuk(2006) 1 Cx 43 DLBC Sx+CT [35]

28 Gonzalez- Cejudo(2006) 1 Cx 26 DLBC Sx+CT [36]

29 Lorusso(2007) 1 Cx 29 DLBC IE Sx+CT [37]

30 Signorelli(2007 ) 1 32 DLBC CT [38]

31 Ferreri (2008 ) 1 Cx 29 DLBC CT+RT [39]

32 Wuntakal(2008) 1 Cx 60 DLBC CT [40]

33 Ab(2008) 1 Cx 43 DLBC IE CT [41]

34 Baijal( 2009) 1 Cx 44 DLBC CT+RT [42]

35 Nazeerahamad(2011) 2 Cx 49 DLBC IEA CT+RT [43]

Cx 51 DLBC IIEA CT+RT

36 Margriet(2011) 1 Ut 79 DLBC CT [44]

37 Parva(2011) 1 Cx 21 DLBC CT [45]

38 Binesh (2012) 1 Cx 85 DLBC CT [46]

39 Parnis (2012) 1 Cx 54 DLBC CT+RT [47]

40 Vasudev (2012) 1 Cx 52 DLBC Sx [48]

41 Mouhajir (2014) 1 Cx 49 DLBC CT+RT [49]

42 Cao 2014 1 20 DLBC CT [50]

43 Runzhe(2015) 1 Ut 63 DLBC IEA CT [51]

44 Posfai (2015) 1 Ut 27 DLBC CT [52]

45 Cubo(2017) 1 Cx 51 DLBC CT [53]

46 Boussios(2018) 1 60 DLBC CT+RT [54]

47 Roberts (2018) 1 Cx 55 DLBC CT [55]

48 Gui (2019) 1 Cx 36 DLBC CT [56]

49 Goda(2020) 3 Cx 52 DLBC CT+RT [57]

Cx 50 DLBC CT+RT

Cx 39 DLBC CT+RT

50 Akkour(2021) 1 54 DLBC Sx+CT+RT [58]

51 Stabile(2022) 1 Cx 83 DLBC I E-A Sx+RT+CT [59]

52 Quaresima (2022) 1 Cx 30 DLBC CT [60]

53 Capsa(2022) 1 Cx 75 DLBC CT+RT [61]

54 Yu-Fei(2023) 1 Ut 83 DLBC CT [62]

55 Present study 1 Ut 73 DLBC IV CT

Total no 141 Cx- 66.9% 45.5 DLBC (76) IE (69) Sx+RT (25)

DLC (27) IIE (23) CT+RT (33)

Histiocytic (13) IIIE (3) Only Sx (13)

Nodular (7) IV (9) Only RT (11)

Follicular (1) Only CT (30)

Burkitts (1) Sx+CT (13)

DSC (5) Sx+CT+RT (4)

DMC (1)

LL (1)

MZL(1)

S.N., Serial number; Tx, treatment; FU, follow up; Cx, cervix; Ut,, uterus; Vg, vagina; BM, bone marrow; LN, lymph node; Sx, surgery; CT, chemotherapy;RT, radiotherapy; DLBC, diffuse large B cell; DLC, diffuse large cell; DMC, diffuse mixed cell; MZL ,marginal zone lymphoma
